# Supplementary material for: Comparative Molecular Dynamics Investigation of the Electromotile Hearing Protein Prestin
Source: Int J Mol Sci. 2021 Aug 2;22(15):8318. doi: 10.3390/ijms22158318 (PMC8347359; doi:10.3390/ijms22158318)
Supplement: Supplementary file 1 [file ijms-22-08318-s001.zip › ijms-1299120-supplementary.pdf]

Comparative Molecular Dynamics Investigation of the Electromotile Hearing Protein Prestin

Gianfranco Abrusci[1,2 †], Thomas Tarenzi[1,2 †], Mattia Sturlese[3], Gabriele Giachin[4], Roberto Battistutta[4] and Gianluca Lattanzi[1,2,*]

[1] *Department of Physics, University of Trento, Italy*

[2] *TIFPA-INFN, Trento, Italy*

[3] *Department of Pharmaceutical and Pharmacological Sciences, University of Padova, Italy*

[4] *Department of Chemical Sciences, University of Padova, Italy*

[†] These authors contributed equally to this work

* Correspondence: gianluca.lattanzi@unitn.it

S1 Validation of the structural models

S1.1 Comparison with SLC26A9 structure

In order to assess the reliability of the protein models and the sampled conformations, rPres and zPres inward structures were aligned to the structure of murine SLC26A9 (PDB ID: 6RTC). The SulTP domain of SLC26A9 is the only structure from an eukaryotic SLC26 member that has been experimentally resolved so far [1]. Table S1 provides RMSD and Template Model score (TM-score) between the structure of SLC26A9 and the simulated configurations giving the best alignment. The TM-score was calculated using the TM-ALIGN web server [2]. It takes values ranging from 0 to 1; values lower than 0.30 indicate a random structural similarity between the input structures, while values in the range 0.5 to 1.0 indicate that the fold pattern is the same.

On the one hand, the high values of the TM-score show that the folded structures are highly similar. On the other hand, RMSD values take into account two main structural

Protein	RMSD (Å)	TM score
rI	3.65	0.80440
zI	3.47	0.79189

Table S1: RMSD and Template Model score of rPres and zPres with respect to 6RTC.

differences, highlighted in Figure S2: SLC26A9 has a short helix buried in the membrane and in contact with TM14, while rI does not show the same arrangement. However, the segment, spanning residue 238 to 258, is highly hydrophobic; therefore it is likely that in longer trajectories it might penetrate the membrane, and that a better agreement might be achieved. However, this helix is not related to the binding of ions, and its orientation will likely not affect the results of the present work. The other major difference is due to the highly flexible loop 151-177, which, being disordered, prevents a thorough comparison with the experimental data. Apart from these differences, the models show a good agreement with the experimental structure.

S1.2 Water accessibility in rI: comparison with experimental data

The calculation of water density and solvent exposure at residue-level was used as validation of the simulations, through direct comparison with experimental data. Figure S3 shows the distribution of water in the cavity of the zPres Inward state, where a continuous flow of water molecules is impaired by hydrophobic residues. Similar results were obtained for the rPres Inward. In the equilibrated part of the trajectories, continuous water passage through the protein is impaired in the the outward states too.

The work by Gorbunov et al. [3] provides a per-residue level of detail for the exposure of amino acids to solvent in mammalian prestin, allowing for a direct comparison with our rPres model. Via the Substituted Cysteine Accessibility Method (SCAM) scan, different residues were selectively mutated into cysteines. Cysteine-reactive reagents like (2-sulphonatoethyl)methane- thiosulphonate (MTSES) and 2-(trimethylammonium)ethyl]-methane-thiosulphonate (MTSET) were used to extract information about the solvent availability and about which part of the membrane each residue was exposed to. All the residues experimentally mutated in cysteines (represented in Figure S4) were shown to be accessible to water. For comparison, the number of water molecules nearby each residue are here calculated during the whole simulated trajectory, assuming a reference distance of 3 Å between the oxygen atoms of water molecules and the atoms of the selected residues. Fig. S5 shows the distribution of the number of water molecules around each specified amino acid; the results from the simulations are in excellent agreement with the experimental data.

The only discrepancy is related to L431. This residue is located at the interface between the protein and the membrane, with its sidechain buried in the hydrophobic core of the protein and its backbone exposed to the solvent. The mutation L431C, necessary to probe its exposure to water, introduces a polar residue that, in contrast, can highly favour interactions with the polar solvent. The nature of the amino acid could therefore induce a better exposure to the solvent than the original residue.

S2 Equilibration

2D RMSD matrices of C^α atoms have been computed to assess equilibration of the starting models, and to investigate the regions of the conformational space explored during the dynamics (Figure S6). For rI, the plot describes two major conformational basins: the first between 50 and 400 ns and the second from 400 ns to the end of the simulation. The first 50 ns represent the equilibration of the system. With respect to rI, zI requires a longer equilibration time (100 ns) before reaching more energetically favourable states.

On the other hand, the trajectories of the outward states can be split in two: around 350 ns for rPres and 300 ns for zPres. In the first 50 ns of the simulations, several tens of permeation events occurred, with water molecules passing through the proteins in a channel-like fashion. Figure S7 shows the path followed by a water molecule in zO. Such solvent permeability is in contrast with the selective solvent accessibility of the putative binding site, as shown in previous experiments [3]; indeed, the process did not occur at any later stages, in the equilibrated simulation. The reason for this is apparent by comparing the first and the last frame of each simulation: TM4 changes its inclination of about 10° with respect to the starting frame, closing the hydrophobic core of the protein and thus blocking the path for solvent molecules.

According to the RMSD matrices, rI shows the sharpest separation between the configurations explored during the dynamics. To further investigate the reason for this behaviour, the per-residue Root Mean Squared Fluctuation (RMSF) was calculated (Figure S8). As expected, the peaks in the RMSF are in correspondence of loop regions, in particular those facing the extracellular side. The latter have been experimentally shown to modulate the electric potential on the membrane surface, thus tuning the operating voltage range of mammalian prestin [4]. The extracellular loop between residues 151 and 177 in rPres Inward is the one presenting the highest fluctuations. Visual inspection of the trajectories reveals that this loop loses interactions with other protein domains, and is then free to fluctuate. In particular, its fluctuations are modulated by the breaking of polar contacts (N163-R236) and hydrophobic interactions (V157-Y313, V157-P108). RMSF plots reveal a striking difference in the flexibility of the second extracellular loop in the two outward systems: in the zebrafish case the fluctuations are largely reduced with respect to the rat protein, because of the tendency of the loop to form a β-turn.

In order to filter out the influence of large loops fluctuations, the all-to-all RMSD was re-computed for residues with RMSF lower than 6 Å for the Inward states, and lower than 4 Å for the Outward ones (Figure S9). By not taking into account the first extracellular loop (residues 151-177), the sharp separations between the conformational states of rI is removed. In the rO case, the filtered RMSD confirms the convergence of the simulation after 200ns. zPres in both states does not show major differences with respect to the full RMSD case, except for an increased separation of the first 100 ns and 300 ns in the inward and outward state, respectively, from the rest of the trajectory.

Protein	Num. Clusters
rI	4
rO	6
zI	7
zO	6

Table S2: Number of clusters per trajectory, as computed using GMM [5]

S3 Additional figures

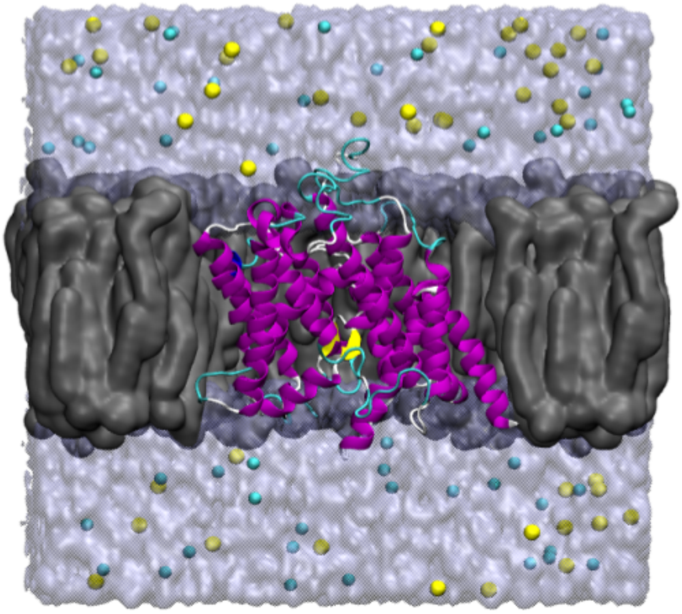

Figure S1: Final setup of rI embedded in the lipid bilayer.

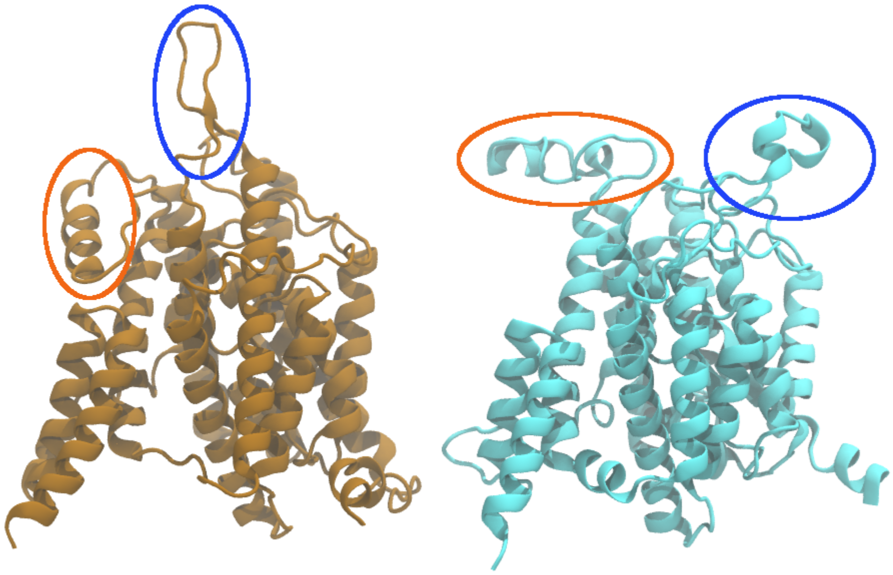

Figure S2: Crystal structure of SLC26A9 (*left*) and model of rPres Inward (*right*). The red and blue circles highlight the different disposition of the short helix (residues 238-258) and the extracellular loop (residues 151-177), respectively.

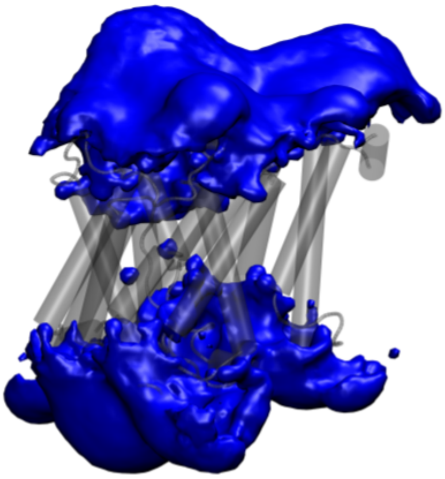

Figure S3: Isosurface of water density around zI structure.

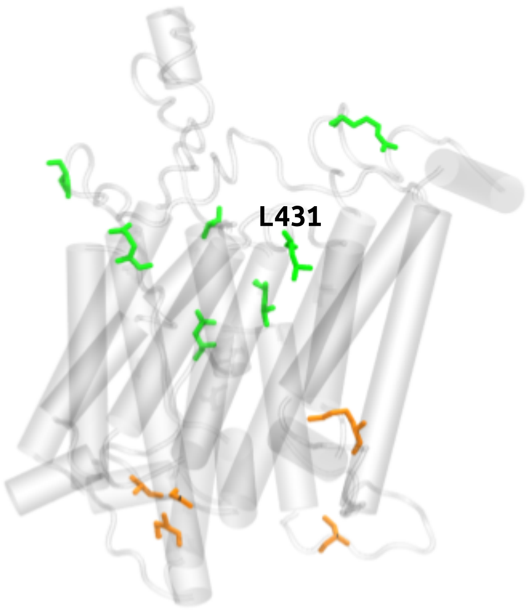

Figure S4: Residues of the extracellular and intracellular protein surface whose solvent exposure has been investigated.

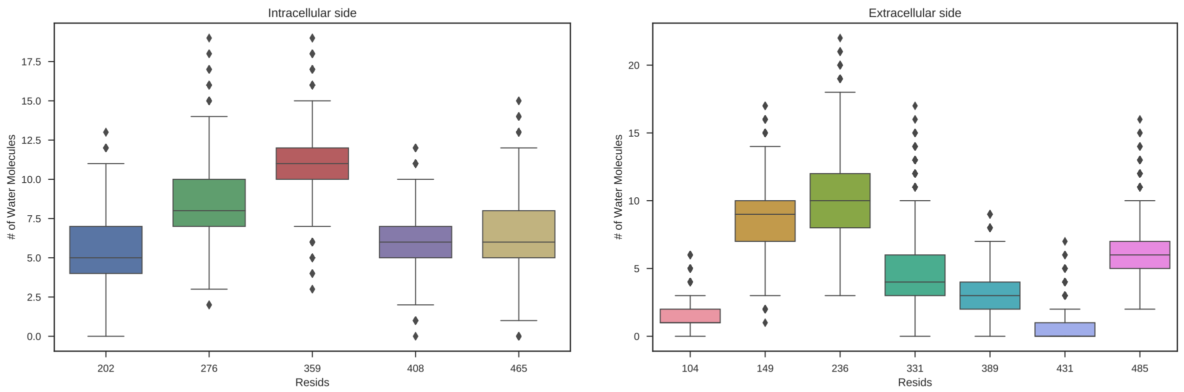

Figure S5: Distributions of the number of water molecules in the proximity of selected prestin residues, calculated along the rPres Inward simulation.

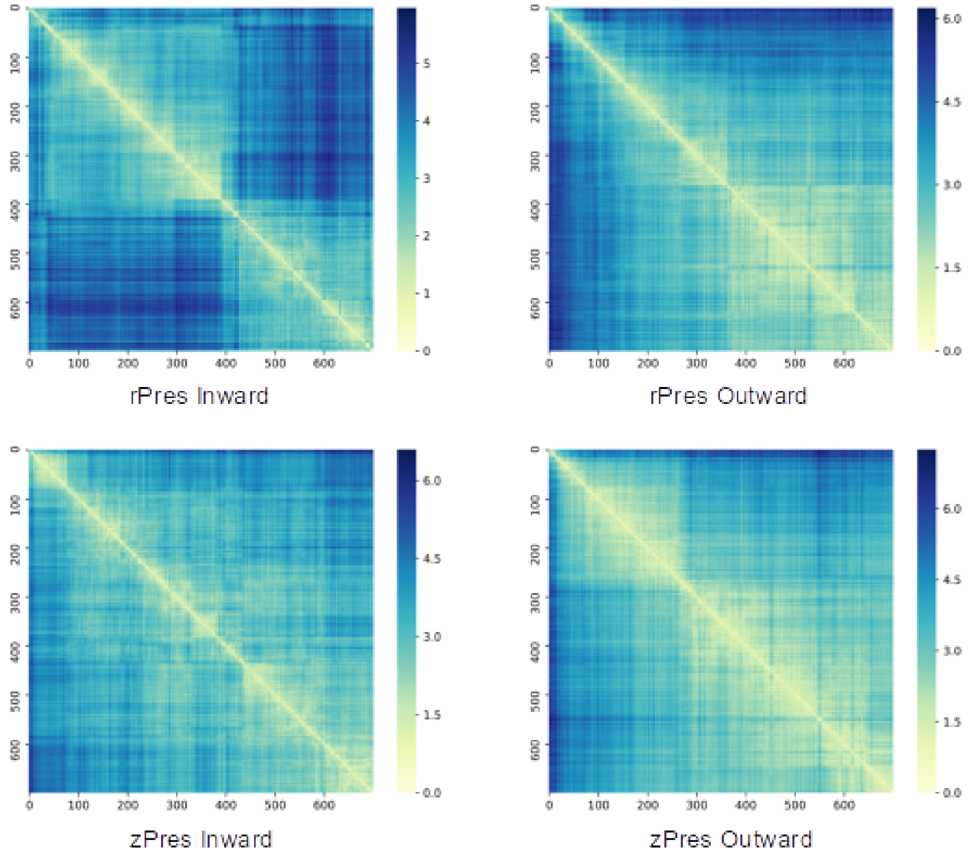

Figure S6: All-to-all RMSD of the four simulations.

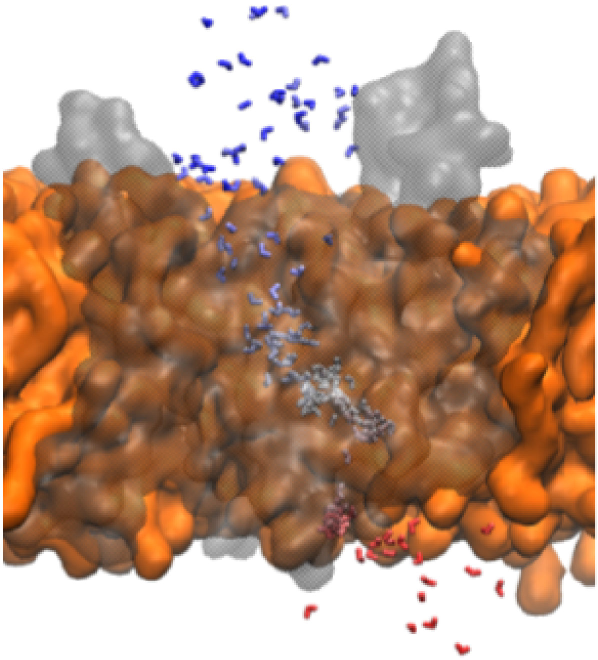

Figure S7: Permeation events occurring in the outward conformation of zPres in the first 50ns of simulation.

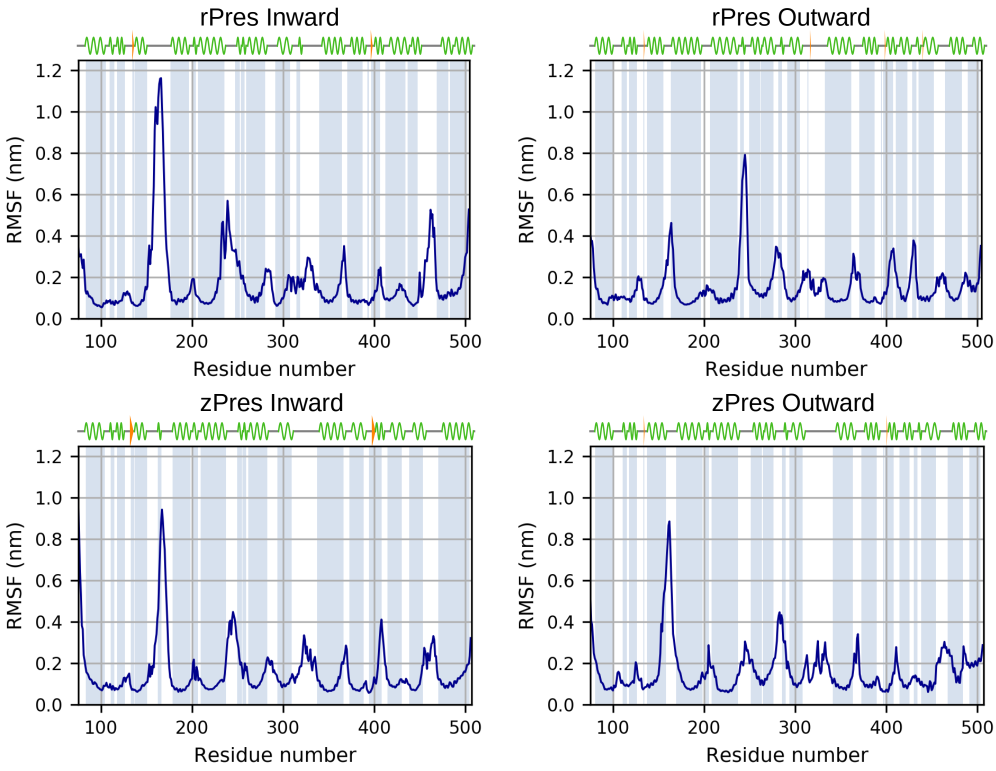

Figure S8: RMSF of the four structures. Shaded areas correspond to structured regions of the proteins, as indicated on top on each plot. The secondary structure of the proteins is depicted using Biotite [6]; green waves represent α-helices, and orange arrows correspond to β-strands.

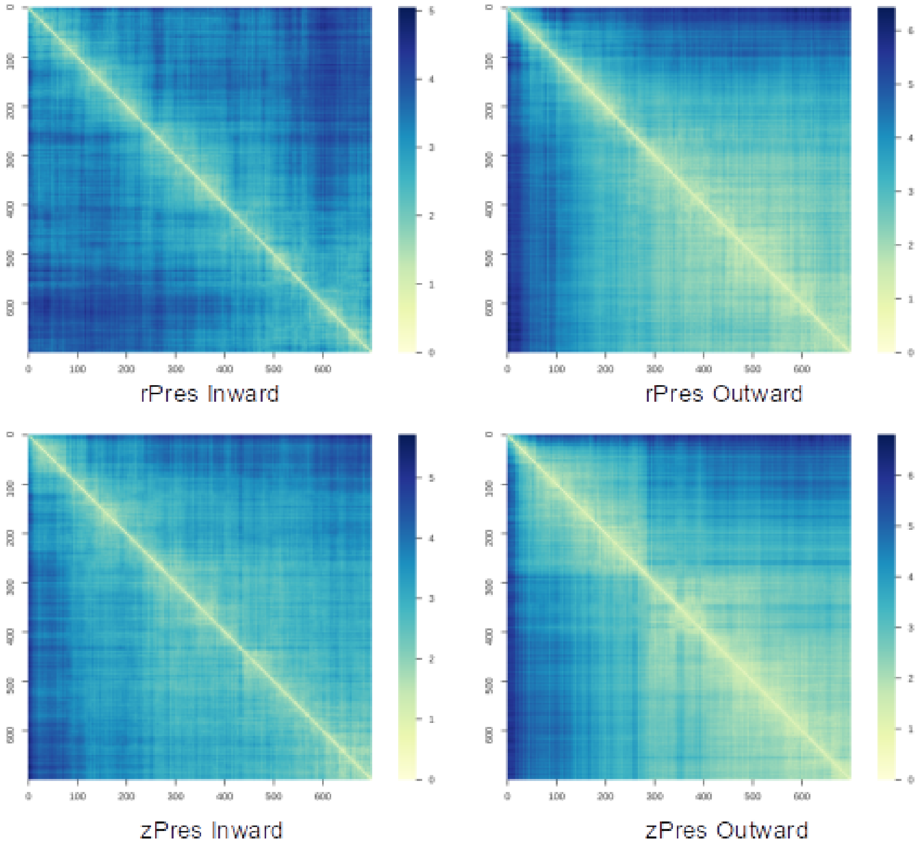

Figure S9: All-to-all RMSD computed on the transmembrane region only.

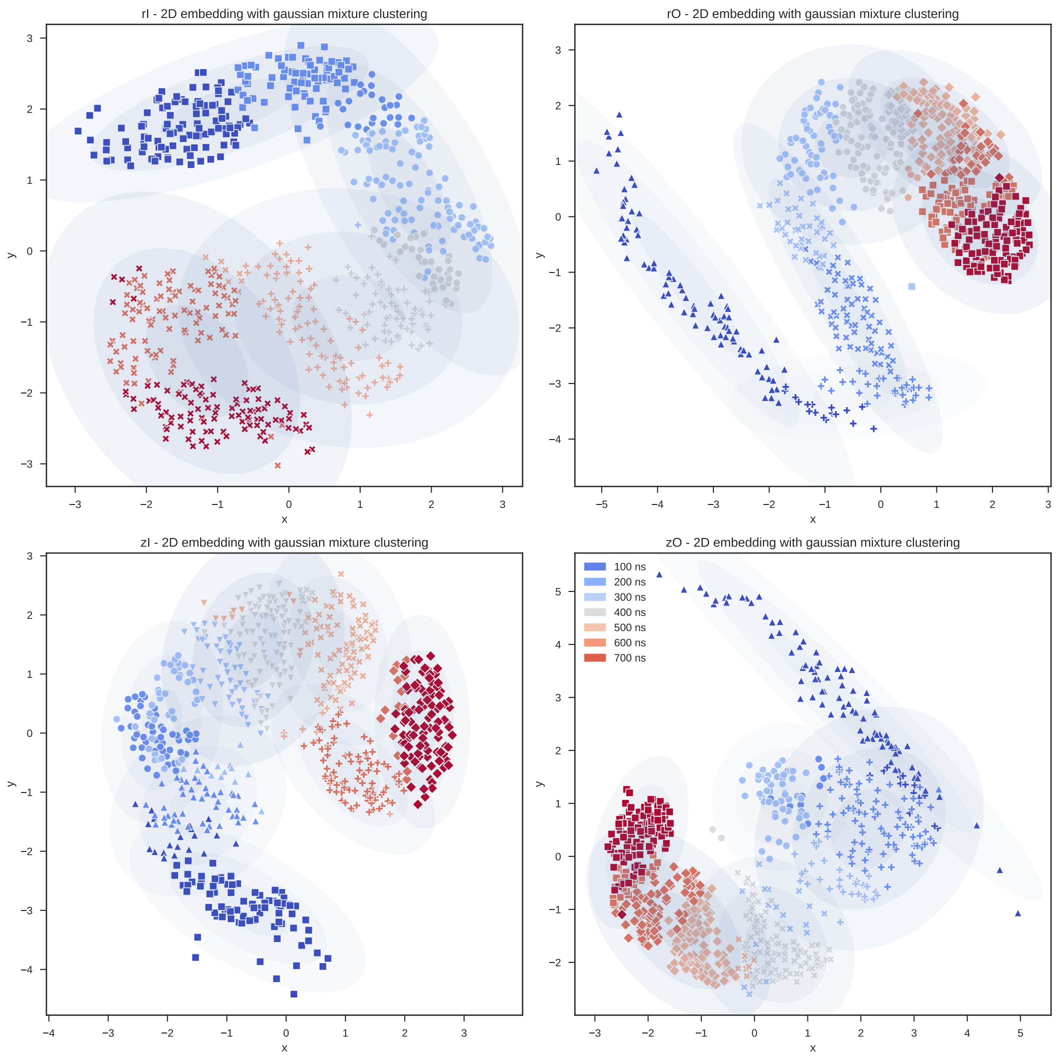

Figure S10: MDS projections with GMM clustering. Top panels: rPres Inward and Outward. Bottom panels: zPres Inward and Outward. Each point corresponds to a frame from the simulation. The markers represent the cluster they belong to, while the shaded areas describe the gaussian probability density computed with the Gaussian mixture model.

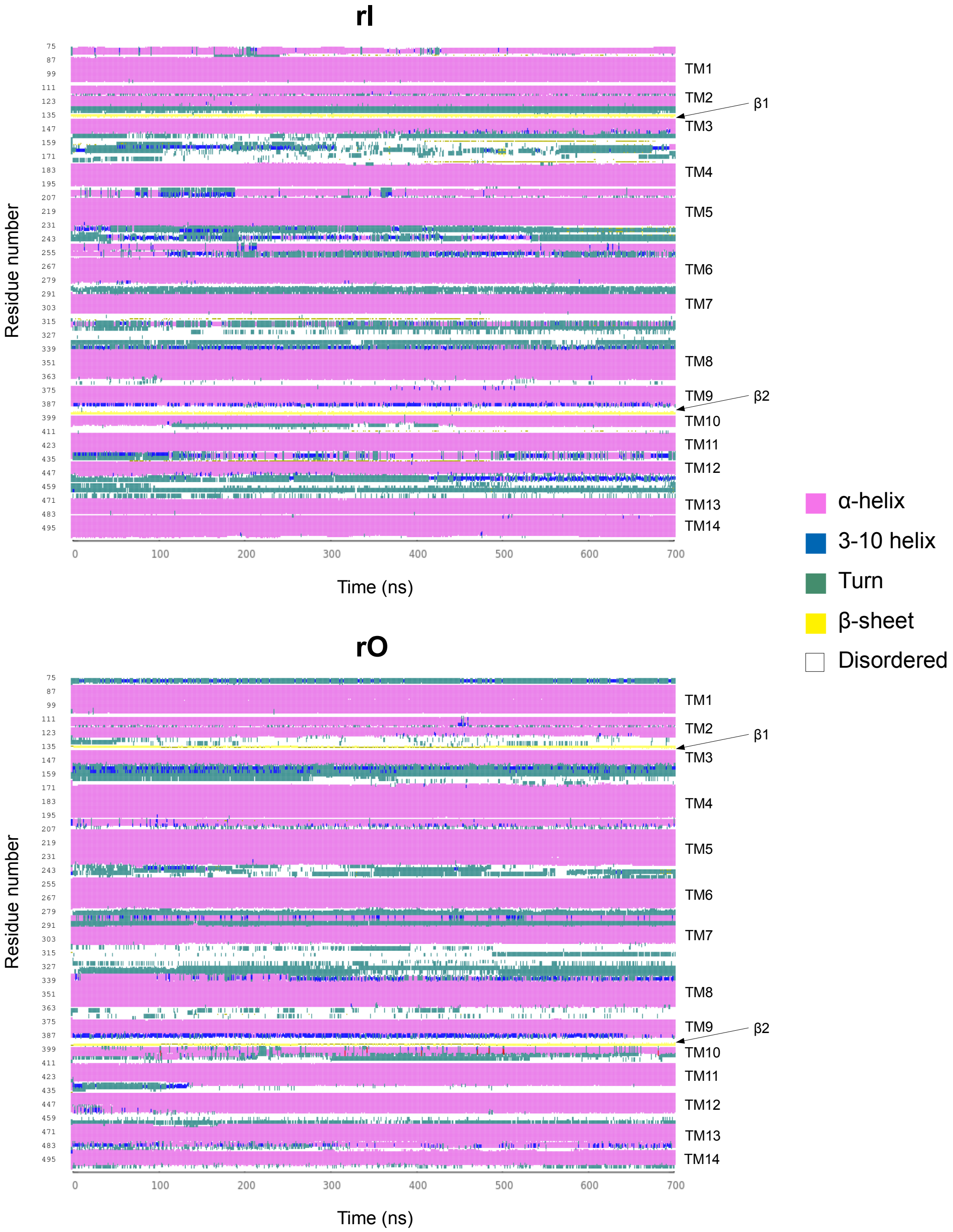

Figure S11: Secondary structure of rI and rO, during the course of the simulations. The indices of the transmembrane helices (TM) are indicated on the right, as well as the two β-strands (β_1 and β_2).

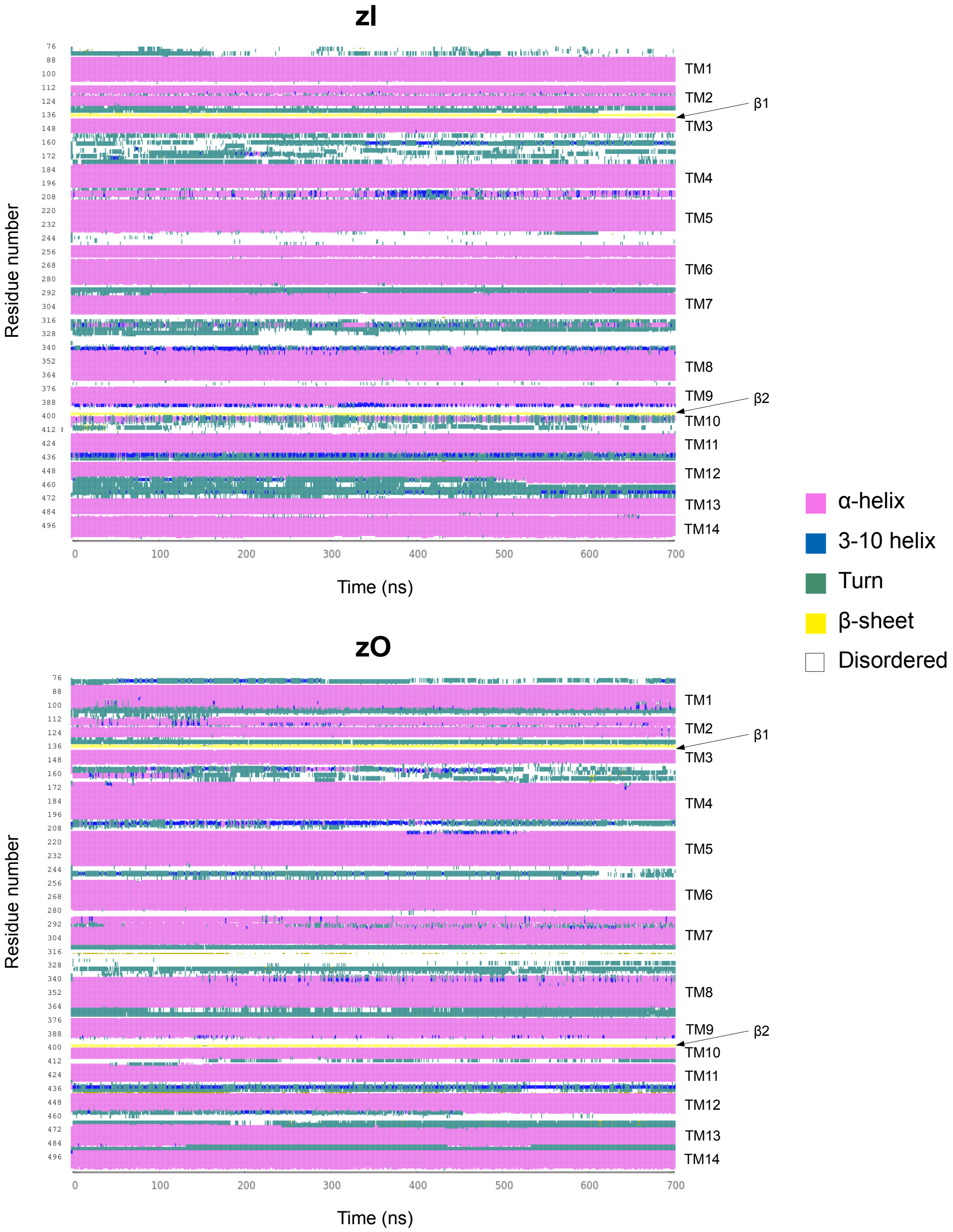

Figure S12: Secondary structure of zI and zO, during the course of the simulations. The indices of the transmembrane helices (TM) are indicated on the right, as well as the two β-strands (β_1 and β_2).

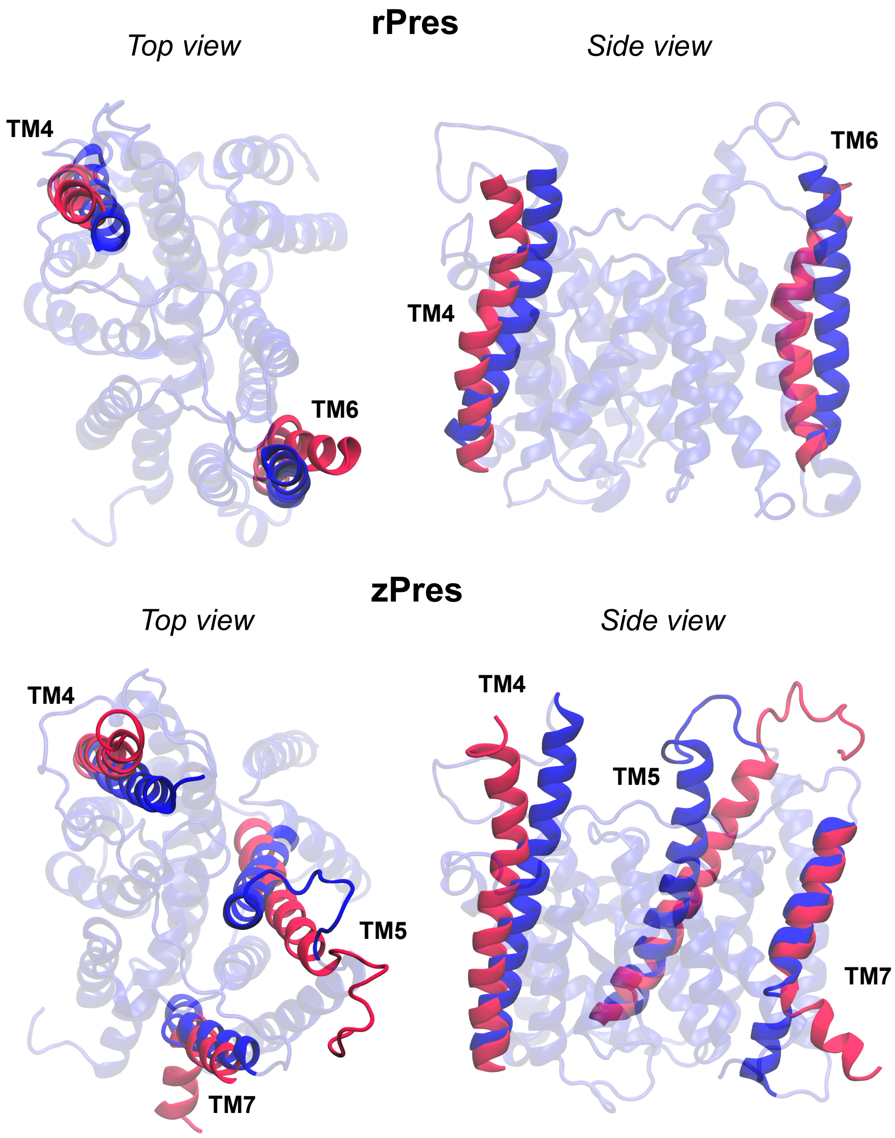

Figure S13: Conformation of the helices undergoing the largest rearrangement from the starting model (*red*) to the equilibrated structure (*blue*) of rPres and zPres in the outward-open conformation.

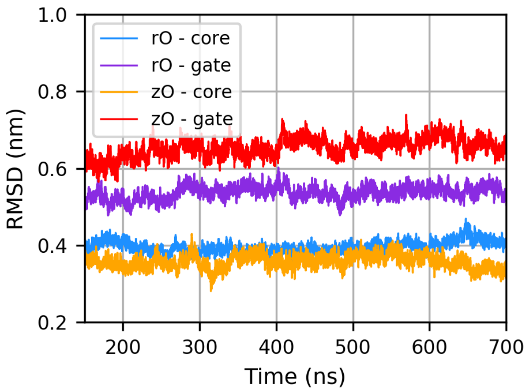

Figure S14: RMSD of the individual domains computed between the conformations sampled in the outward-open simulations and the most representative structure in the inward state.

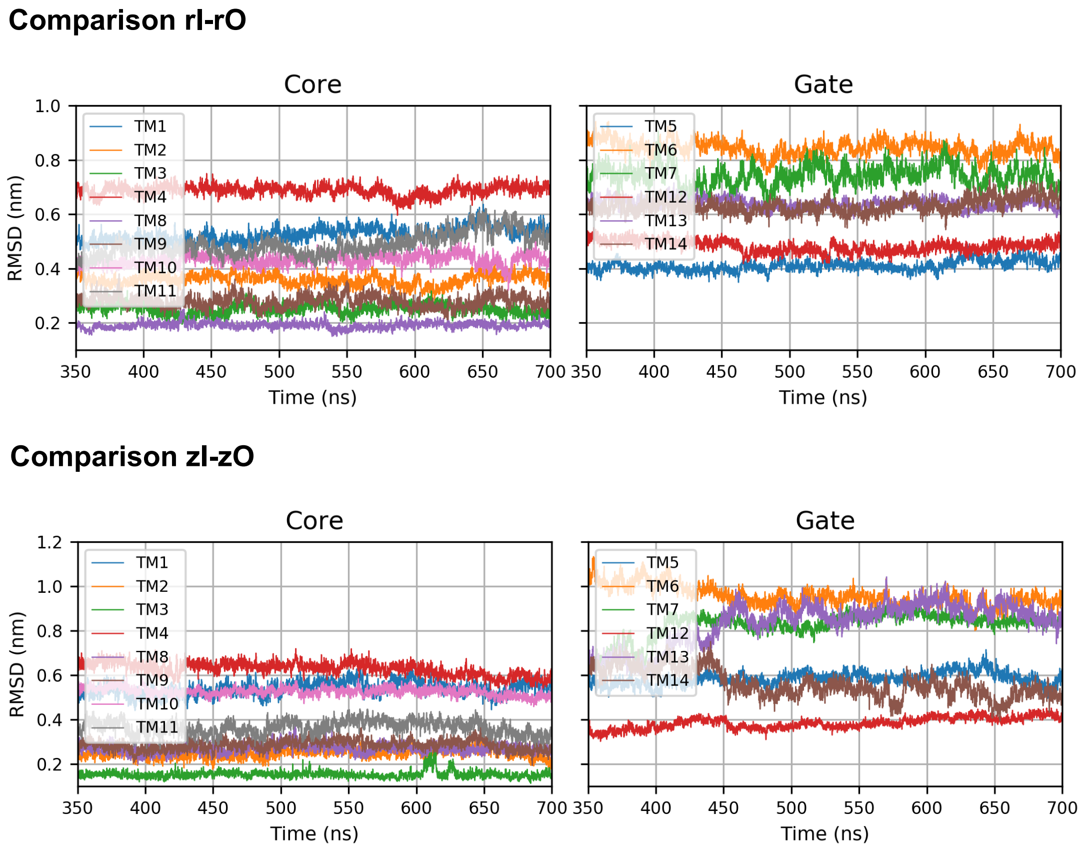

Figure S15: RMSD computed on each transmembrane helix (TM) between the conformations sampled in the outward-open simulations and the most representative structure in the inward state, after structural alignment of the core/gate domains, according to the location of the TM.

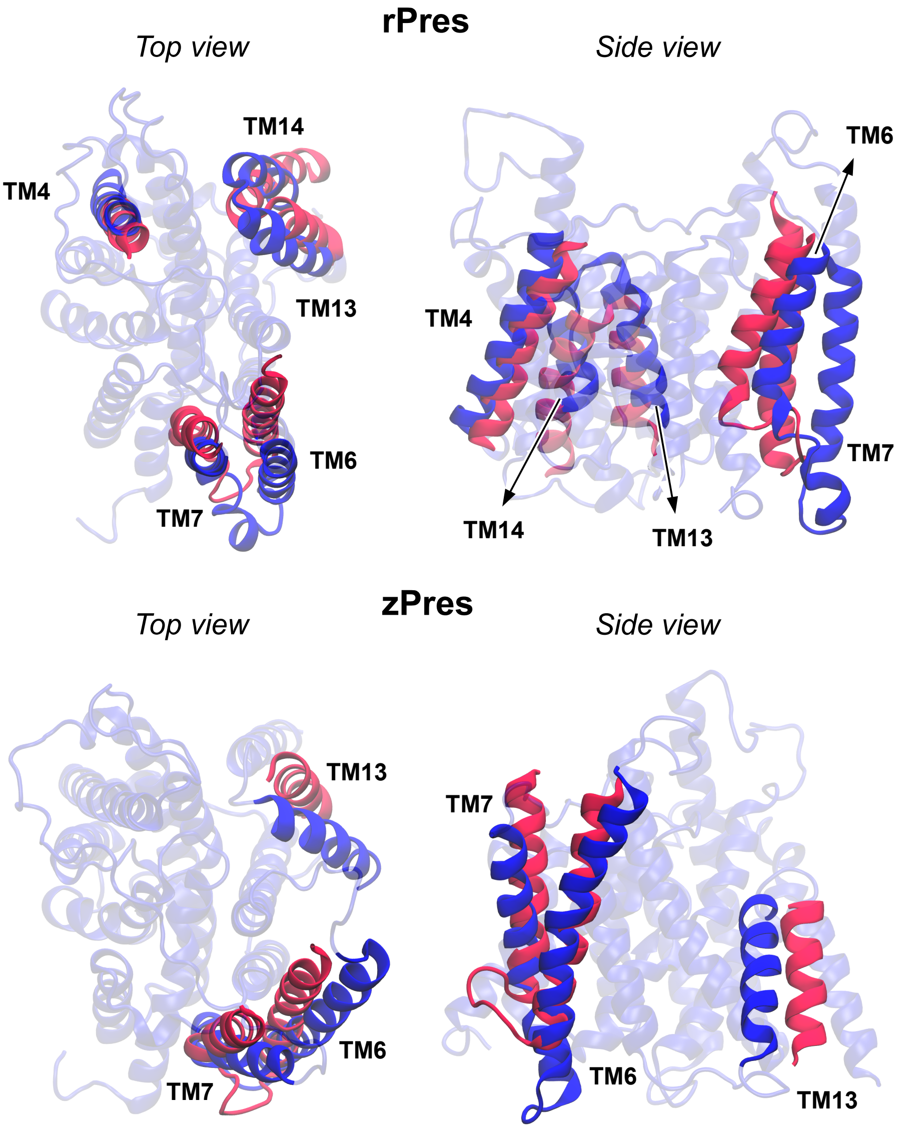

Figure S16: Conformation of the helices undergoing the largest rearrangement when comparing the inward-open conformations (*red*) and the outward-open ones (*blue*), for both rPres and zPres.

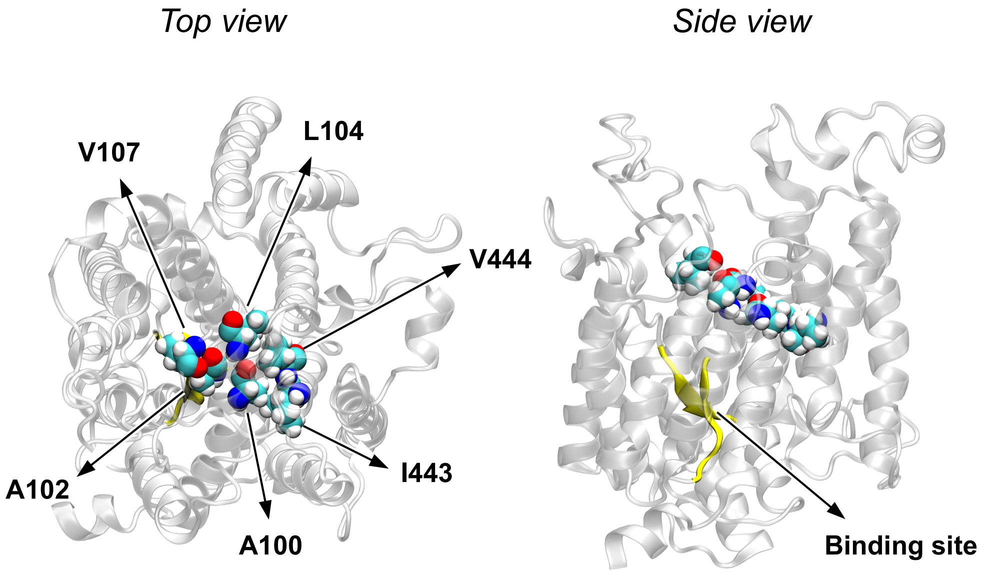

Figure S17: Hydrophobic cluster positioned above the binding site (in yellow) in rI. The residues belonging to the cluster are represented as Van der Waals spheres.

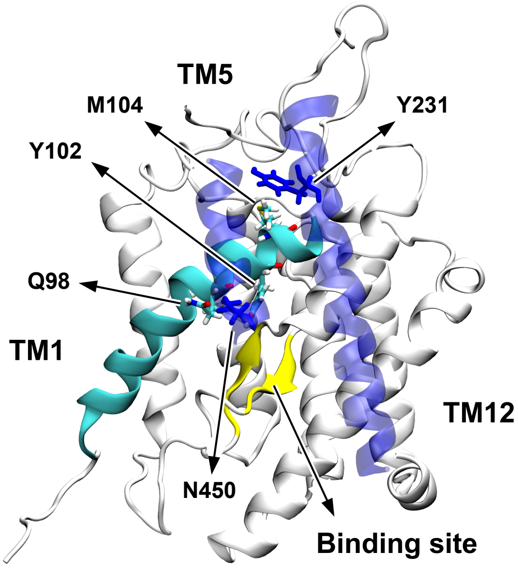

Figure S18: Position of TM1 (cyan) above the binding site (yellow) in zI, stabilizied by interactions with TM5 and TM12 (blue).

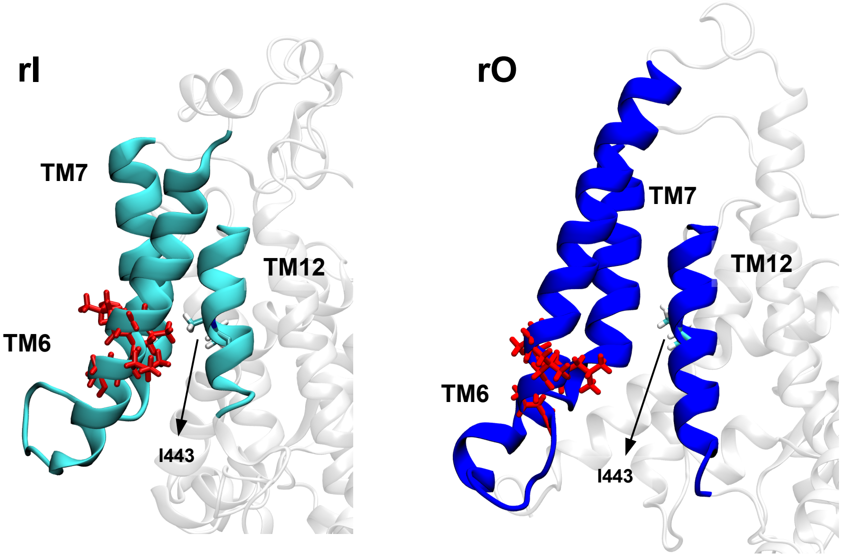

Figure S19: Relative positions of TM6 and TM7 with respect to the inner helix TM12. In rI, the contact is stabilized through the conserved region 270-275 (in licorice, red), in particular by the interaction L272-I443. In rO, TM6 and TM7 loose the contact with TM12 and extend through the membrane.

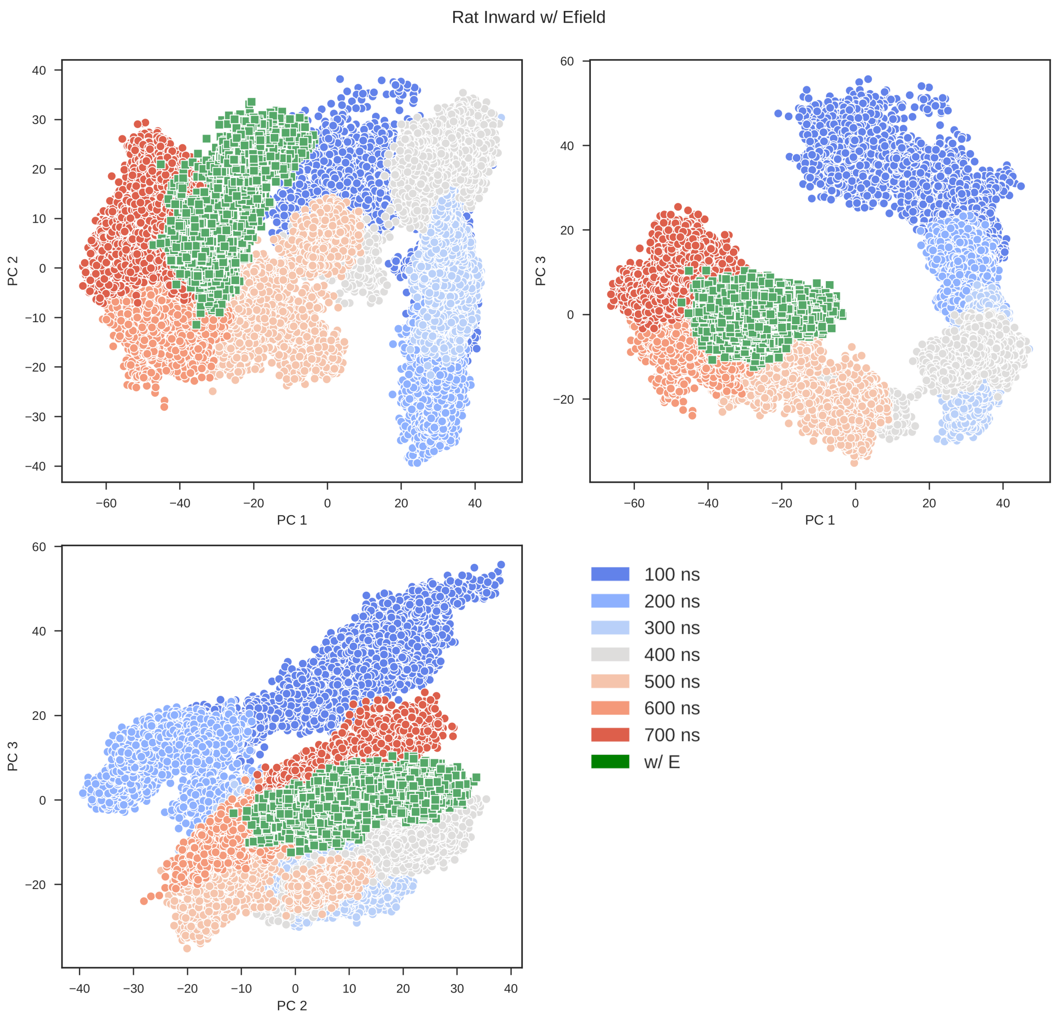

Figure S20: PCA of rPres Inward with the electric field applied, compared to the simulation where the electric field is absent.

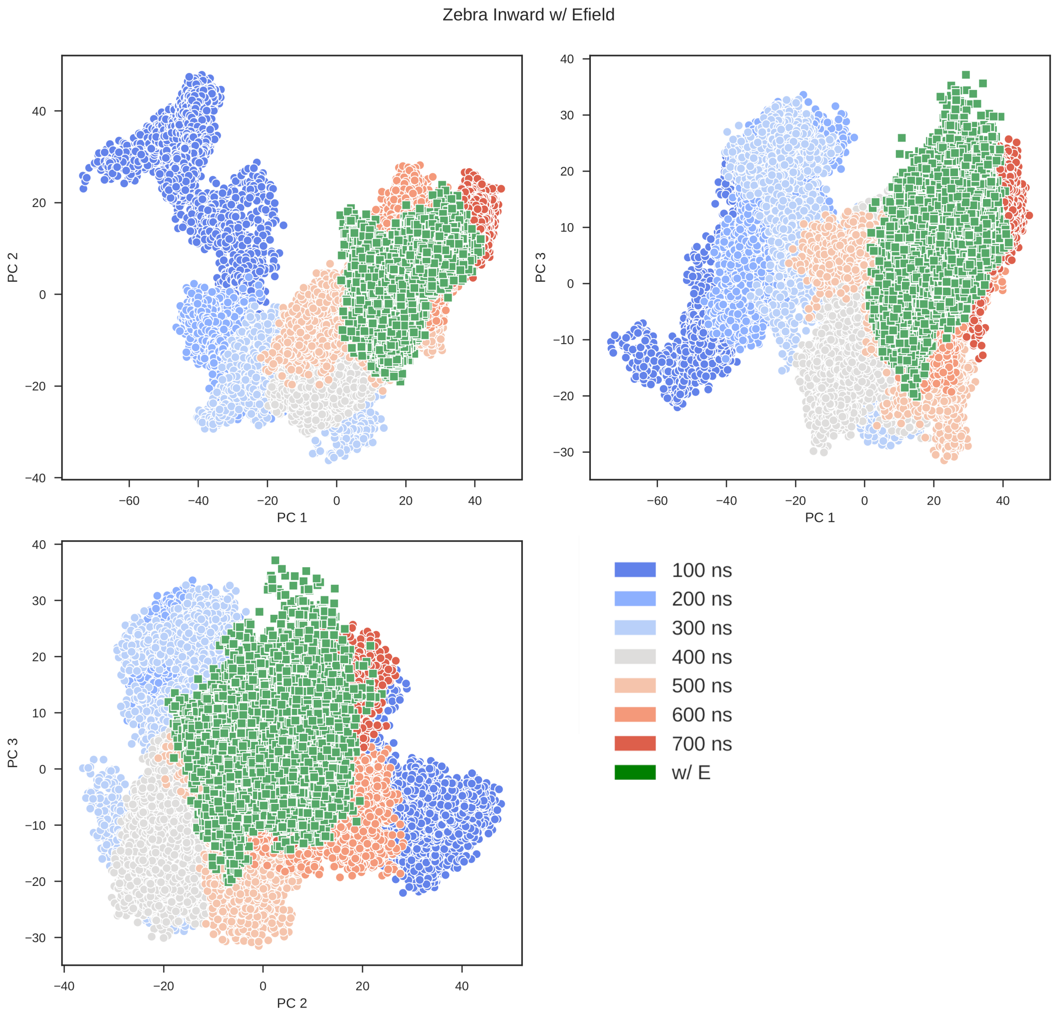

Figure S21: PCA of zPres Inward with the electric field applied, compared to the simulation where the electric field is absent.

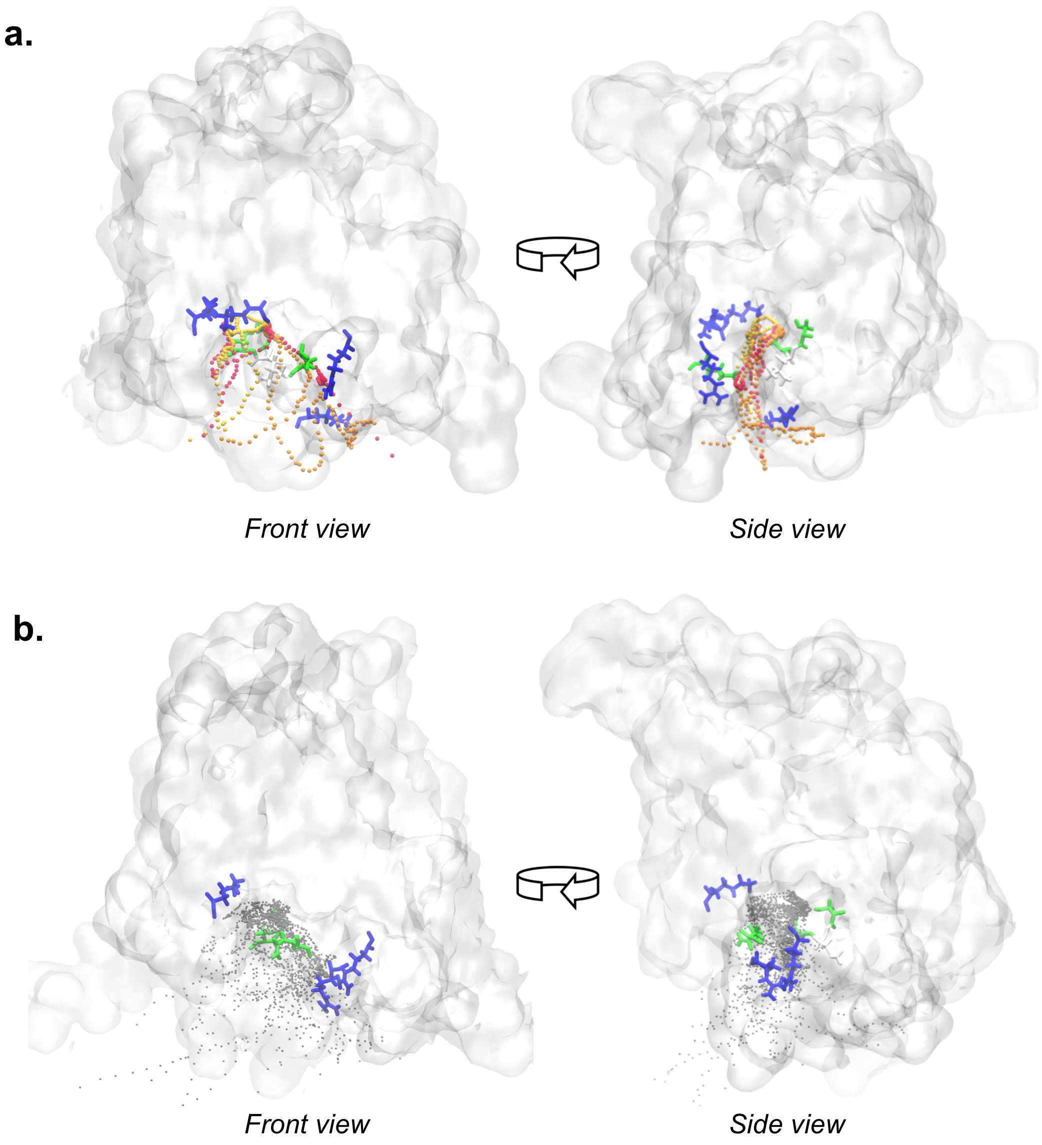

Figure S22: Front and side views of the ion pathways in the putative binding site of rI (**a**) or zI (**b**) when the electric field is switched on.

References

[1] J. D. Walter, M. Sawicka, and R. Dutzler, “Cryo-em structures and functional characterization of murine slc26a9 reveal mechanism of uncoupled chloride transport,” *Elife*, vol. 8, p. e46986, 2019.

[2] Y. Zhang and J. Skolnick, “TM-align: a protein structure alignment algorithm based on the TM-score,” *Nucleic Acids Research*, vol. 33, no. 7, pp. 2302–2309, 2005.

[3] D. Gorbunov, M. Sturlese, F. Nies, M. Kluge, M. Bellanda, R. Battistutta, and D. Oliver, “Molecular architecture and the structural basis for anion interaction in prestin and SLC26 transporters,” *Nature Communications*, vol. 5, p. 3622, 2014.

[4] M. F. Kuwabara, K. Wasano, S. Takahashi, J. Bodner, T. Komori, S. Uemura, J. Zheng, T. Shima, and K. Homma, “The extracellular loop of pendrin and prestin modulates their voltage-sensing property,” *Journal of Biological Chemistry*, vol. 293, no. 26, pp. 9970–9980, 2018.

[5] G. Yu, G. Sapiro, and S. Mallat, “Solving inverse problems with piecewise linear estimators: From gaussian mixture models to structured sparsity,” *IEEE Transactions on Image Processing*, vol. 21, no. 5, pp. 2481–2499, 2011.

[6] P. Kunzmann and K. Hamacher, “Biotite: a unifying open source computational biology framework in python,” *BMC bioinformatics*, vol. 19, no. 1, pp. 1–8, 2018.
